# Effect of Toughening with Different Liquid Rubber on Dielectric Relaxation Properties of Epoxy Resin

**DOI:** 10.3390/polym12020433

**Published:** 2020-02-12

**Authors:** Chuang Wang, Qing Sun, Kang Lei, Chi Chen, Lixiao Yao, Zongren Peng

**Affiliations:** 1 School of Electrical Engineering, Xi’an University of Technology, Xi’an 710048, China; 2 State Key Laboratory of Electrical Insulation and Power Equipment, Xi’an Jiaotong University, Xi’an 710049, China

**Keywords:** epoxy resin, liquid rubber, polarity, interfacial polarization

## Abstract

Liquid rubber is a common filler introduced to epoxy resin to improve its toughness for electrical insulation and electronic packaging applications. The improvement of toughness by adding liquid rubber to epoxy resin leads to the variation of its dielectric properties and relaxation behaviors and it has not been systematically studied yet. In this paper, four kinds of liquid rubber with different polarity were selected and the corresponding epoxy/liquid rubber composites have been prepared. By analyzing the temperature and frequency dependence of dielectric spectra, we found that a lower relative dielectric constant and dielectric loss of the epoxy/liquid rubber composites could be achieved by reducing the polarity of liquid rubber filler. These results also confirm that the polarity of liquid rubber plays a critical role in determining the α transition relaxation strength of rubber molecules at about −50 °C, as well as the relaxation time of interfacial polarization. In addition, the conductivity of rubber phase with different polarity were investigated by studying the apparent activation energy of interfacial polarization calculated from the Arrhenius plot. This study can provide a theoretical basis for designing high-performance epoxy/liquid rubber composite insulating materials for industrial use.

## 1. Introduction

Epoxy resin is widely used as insulation material and adhesives in power equipment and electronic packaging [1,2]. The cured epoxy resin is brittle and prone to brittle facture. Thus, fillers are added into epoxy resin to enhance its toughness for certain applications. The type of fillers added to epoxy resin normally consists of inorganic particles, organic particles, thermoplastic resins, and liquid rubbers [3,4]. Liquid rubber fillers are one of the common choices for industrial applications as it is more economical and easier to be introduced into epoxy resin [5,6].

After adding liquid rubber, epoxy resin and rubber are existing as a two-phase structure in the cured epoxy resin composites [7]. The liquid rubber functioned as the toughening phase mainly consists of the derivative of polybutadiene. The commonly used liquid rubbers are carboxyl-terminated liquid nitrile-butadiene rubber (CTBN) [8], hydroxyl-terminated liquid nitrile-butadiene rubber (HTBN) [9], and hydroxyl-terminated liquid polybutadiene (HTPB) [10]. In addition to toughening epoxy resins, liquid rubber will also affect other properties such as glass transition temperature and conductivity of matrix. For electrical insulation, understanding the dielectric properties and relaxation behaviors of the toughened epoxy resins is of great significance.

Despite a vast literature concerned on the mechanical properties of the toughened epoxy resins, a growing attention has been drawn to its dielectric properties in recent years [11,12,13,14,15,16,17]. Better properties in toughness, thermal conductivity, and breakdown strength were achieved from a ternary copolymer, consisting of epoxy matrix, micron hexagonal boron nitride(h-BN), and CTBN [17]. In addition, much attention has been paid to the blending rubber, such as triblock copolymer poly(styrene)-block-poly(butadiene)-blockpoly(methylmethacrylate) (SBM), for better performance in mechanical and thermal [18]. Previous studies mainly focused on enhancing the dielectric properties such as permittivity, in which the relative dielectric constant (ε′) and loss (tan*δ*) of toughened materials were measured and analyzed. However, little is known about how liquid rubber influence the dielectric properties of epoxy resin. The variations of epoxy resin matrix used in different research makes it difficult for comparison. For example, in reference [11] and [16], the *ε*′ and tan*δ* of epoxy resin will increase with the addition of certain types of liquid rubbers, but decrease with other types. The main factors affecting *ε*′, tan*δ*, and other dielectric properties have not been studied and the selection paradigm of liquid rubbers in designing composite materials has not been clarified. Therefore, it is necessary to compare the dielectric properties of epoxy resin toughened with different liquid rubbers in order to understand the mechanisms behind it.

The interfacial polarization could be introduced by the addition of liquid rubber [16] which will affect the *ε*′ and tan*δ*. Different liquid rubbers have different interfacial polarizations due to their different *ε*′ and conductivity (*σ*). The effect of different liquid rubbers on interfacial polarization has not been investigated in previous research. In addition, the mechanisms of the interfacial polarization formed between the rubber phase and epoxy matrix remains unclarified.

In what follows we introduce four kinds of liquid rubbers with different polarity and functional groups to epoxy resin: HTPB, epoxidized hydroxyl-terminated liquid polybutadiene (EHTPB), CTBN and HTBN. The DC resistivity and dielectric spectra these epoxy/liquid rubber composites mentioned above are measured. By comparing these properties of epoxy resin toughened by those four kinds of liquid rubbers, the effect and mechanism of different polarity liquid rubber on the DC resistivity, *ε*′, and tan*δ* of matrix are analyzed. The variation law and mechanism of interfacial polarization caused by different polarity liquid rubbers are discussed based on their relaxation characteristics. The results of our research can provide theoretical basis for a better selection of liquid rubber fillers for designing high-performance epoxy/liquid rubber composite insulating materials.

## 2. Materials and Samples Preparation

### 2.1. Materials

**Figure 1:**
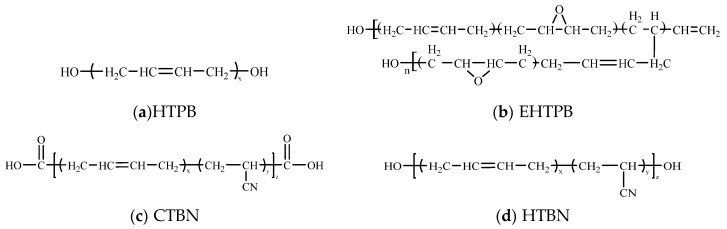
Molecule structures of the four kinds of liquid rubbers. (**a**) hydroxyl-terminated liquid polybutadiene (HTPB); (**b**) epoxidized hydroxyl-terminated liquid polybutadiene (EHTPB); (**c**) carboxyl-terminated liquid nitrile-butadiene rubber (CTBN); (**d**) hydroxyl-terminated liquid nitrile-butadiene rubber (HTBN).

The epoxy resin used in this study is diglycidyl ether of bis phenol A (DGEBP A) with the epoxy value of 4.4 mmol/g produced by Nantong Xingchen Synthetic Material Co., Ltd., Nantong, China. The curing agent is methylhexahydrophthalic anhydride (MeHHPA) produced by Puyang Huicheng Electronic Materials Co., Ltd., Puyang, China. The molecular structures of four kinds of liquid rubbers introduced into epoxy resin are shown in Figure 1.

The hydroxyl content of HTPB is 0.89 mmol/g. The hydroxyl and epoxide content of EHTPB is 0.35 and 1.84 mmol/g, respectively. The carboxyl content of CTBN is 0.61 mmol/g, and the acrylonitrile content is 8.4%. The hydroxyl and acrylonitrile content of HTBN is 0.60 mmol/g and 13.1%, respectively. All the reagents are directly used without treatment.

HTPB is a kind of derivative of butadiene polymer with one hydroxyl functional group on both ends. Due to low polarity and strength of HTPB, it is often modified before use. EHTPB is prepared by epoxidation of HTPB. Additionally, it has greater polarity than HTPB which can be used in the curing reaction of epoxy resin. HTBN is obtained by adding an acrylonitrile segment to the main chain of HTPB [10]. Due to the introduction of nitrile group (–CN), HTBN has some combined properties associated with HTPB and nitrile group. The polarity of HTBN also becomes greater because of the modification of the main chain. The molecular structure of CTBN is similar to HTBN, and only the groups at both ends of the molecule are replaced by carboxyl-terminated groups. Therefore, it has similar properties to HTBN but is easier to react with epoxy resin. HTBN and CTBN are more compatible with epoxy resin due to their greater polarity [19,20]. The content of nitrile group in the selected CTBN is less than that of HTBN, so the polarity of its molecule is slightly smaller than that of HTBN. The polarity of molecular chains of these four liquid rubbers, HTPB, EHTPB, CTBN, and HTBN, increase in turn.

### 2.2. Sample Preparation

Firstly, epoxy resin and liquid rubber are mixed in proportion and pre-crosslinked at high temperature for 1 h in nitrogen atmosphere. Low speed stirring is employed during the crosslinking process. Curing agent and accelerator are added in proportion and vacuum degassing is carried out after mixing. The mixture is then injected into the mould and put into the oven. The curing process is 60 °C/1 h, 80 °C/2 h, 120 °C/4 h, and 160 °C/4 h. The temperature is gradually lowered to room temperature after the curing process.

HTBN, CTBN, and EHTPB are mixed with epoxy resin at 150 °C for pre-mixing. While HTPB needs to be mixed with an accelerator tri-(dimethylaminomethyl) phenol (DMP-30) at 130 °C epoxy/liquid rubber composites with liquid rubber content of 10, 15, 20, 25 phr (per 100 g of epoxy resin) are prepared using those four kinds of liquid rubber mentioned above.

### 2.3. Performance Measurement

The glass transition temperatures of epoxy resins toughened by different liquid rubbers are measured by METTLER DSC822e. The temperature range of measurement is from 30 to 200 °C with a heating rate of 10 °C/min.

The samples are immersed in liquid nitrogen for 2 min and then broke by a clamp into smaller pieces with cut surfaces. Those sample pieces are coated with gold by ion sputtering for scanning electron microscope (SEM) to observe their microstructure. The distribution of four kinds of liquid rubber in the resin matrix and the development of cracks could be observed. The SEM used in our manuscript is VE-9800S, in which the electron gun voltage was set to 20 kV.

KEITHLEY 6517B electrometer and 8009 resistivity test fixtures are used to measure the 60 s DC resistivity of the samples at room temperature around 25 °C. The voltage applied in our measurement is 500 V and the sample size is *Φ*100 mm with a thickness of 1 mm.

The German Novocontrol company’s concept80 broadband dielectric spectroscopy tester is used to measure the dielectric properties. The size of sample is *Φ*40 mm with a thickness of 1 mm. Both surfaces of the samples are coated with gold by the ion sputtering with one side entirely coated but the other within a circle region of *Φ*30 mm. The frequency range and the temperature in this testis from 10^−1^ to 10^6^ Hz from −60 to 200 °C, respectively.

## 3. Influence of Four Kinds of Rubbers on Glass Transition Temperature

**Figure 2:**
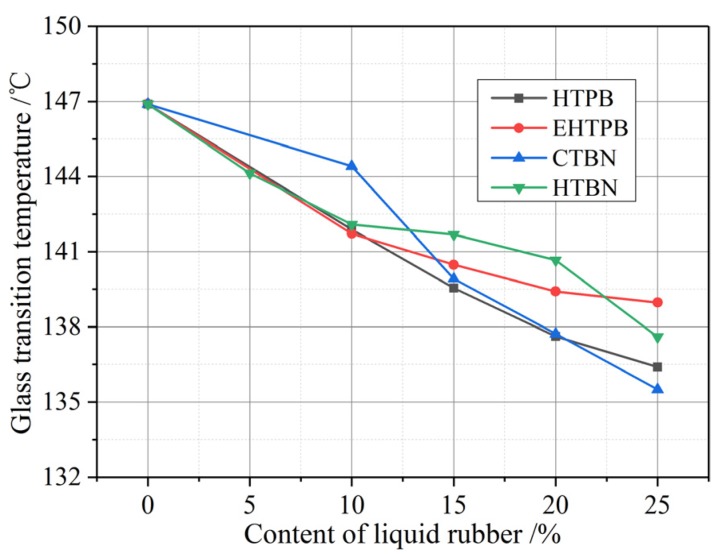
The glass transition temperature of epoxy resin matrix varies with the content of liquid rubber. The glass transition temperature decreases slightly with the increase of liquid rubber content.

The properties of material will change dramatically before and after the glass transition, so the glass transition temperature affects the application temperature range of materials [21]. The effects of different liquid rubber fillers on glass transition temperature of the epoxy/liquid rubber composite are shown in Figure 2.

With the increase of liquid rubber content, the glass transition temperature of all four kinds of samples slightly decrease. When the filler content reaches 25%, the maximum decrease of glass transition temperature is 12 °C (CTBN). Therefore, when the content of liquid rubber is less than 25%, its effect on the application temperature range of composites is not that significant.

The presence of discrete rubber phases hinders the formation of cross-linking structure of epoxy resin, thus reducing the degree of crosslinking. Lower crosslinking degree increases the free volume of material and makes it easier for movement of the chain segments, leading to the decrease of glass transition temperature. The influence of four kinds of liquid rubbers on glass transition temperature has little differences according to Figure 2—the four curves share a same trend and a similar decrease range. This is because those four kinds of liquid rubber form the same two-phase structure with epoxy resin and they have the same effect on the structure of matrix.

The glass transition temperature (*T*_g_) reflects the difficulty of the glass transition process of epoxy matrix. This phase transition process of epoxy resin leads to a relaxation process called α relaxation due to the orientation of epoxy resin segments in the dielectric spectrum. The lower *T*_g_ makes it easier for the segmental chain to move, thus decreases the relaxation time of α relaxation. As the temperature increases, the α relaxation with lower *T*_g_ is prone to move to the high frequency region. As mentioned before, *T*_g_ decreases slightly with the increase of liquid rubber content. Therefore, only little change of the relaxation time could be found with the variation of liquid rubber contents.

## 4. Microstructure and Microcrack Development

**Figure 3:**
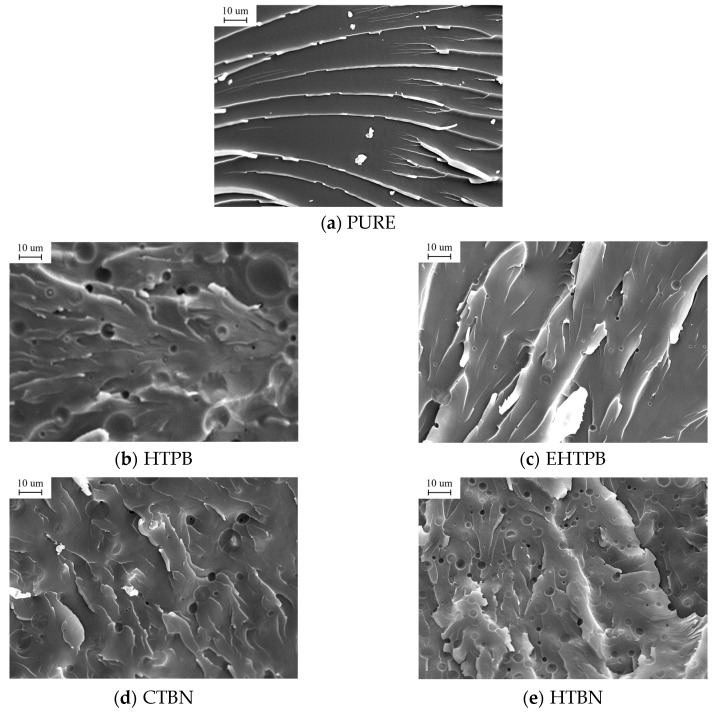
SEM photographs of epoxy resins toughened by different liquid rubbers. (**a**) PURE; (**b**) HTPB; (**c**) EHTPB; (**d**) CTBN; (**e**) HTBN. Liquid rubber exists in the cured epoxy resin as discontinuous rubber particles.

The toughening effect of liquid rubber on epoxy resin mainly depends on the formation of rubber particles in epoxy resin matrix. Therefore, it is necessary to study its microstructure and analyze the state of different liquid rubbers in epoxy matrix. The SEM photos of different samples are shown in Figure 3.

Without adding liquid rubber, most of the cracks are lying across the whole section of epoxy resin (Figure 3a), indicating a typical brittle fracture. As a contrast, the liquid rubbers were introduced to epoxy resin form spherical-shaped rubber particles in the cured samples. The size of rubber particles shows a bimodal or triple-modal distribution. The existence of rubber particles hinders the development of cracks and enhances the toughness of the material. For the samples with HTBN and CTBN, the distribution of rubber particles is more uniform, due to their better compatibility with epoxy resin. Similarly, the poor compatibility with epoxy resin of HTPB makes the HTPB particles show a less uniform distribution. In other words, the size of the separated rubber particles changes more widely. At the same filling content, the number of rubber particles precipitated from the sample with EHTPB is significantly less than that from HTBN and CTBN. This may be attributed to possible occurrences of reactions between EHTPB and its surrounding environment during the curing process, which consumes some proportion of EHTPB particles for precipitation.

Therefore, the polarity of liquid rubber affects the quantity and size of precipitated rubber particles. The liquid rubber with a slightly larger polarity is more compatible with epoxy resin and the size of precipitated rubber particles shows a more uniform distribution.

## 5. Influence of Four Kinds of Liquid Rubber on Dielectric Properties

### 5.1. Analysis of DC Resistivity

**Figure 4:**
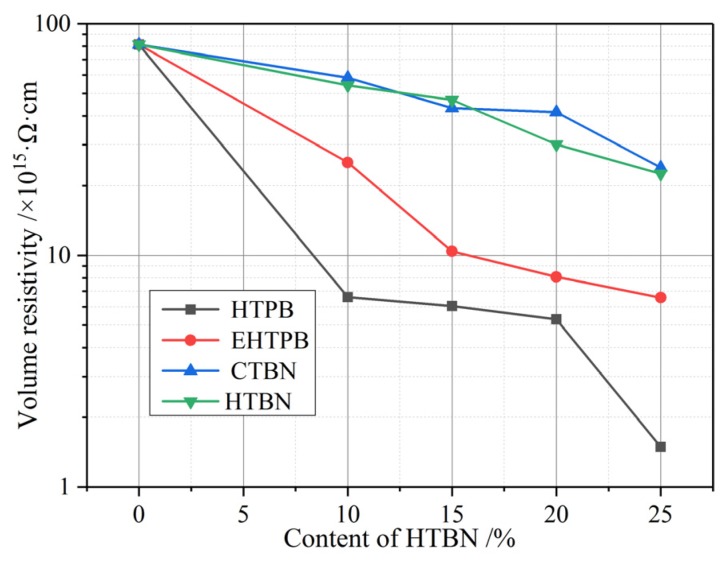
The volume resistivity of epoxy resin matrix varies with the content of liquid rubber. The volume resistivity decreases with the increase of liquid rubber content.

DC resistivity is one of the important parameters of insulating materials. The relevant experimental results are shown in Figure 4.

For all the samples, DC resistivity decreases with the increase of the filler content. The maximum decrease of the DC resistivity is in an order of magnitude when the content of liquid rubber reaches 25%, indicating a trivial effect of liquid rubber fillers on the insulation performance of the composites. The factors that determine the conductivity are the quantity and the mobility of impurity ions [22]. With the addition of liquid rubber, more impurity ions which are proportional to filler contents are introduced. In addition, the free volume in the composites increases with the filler content, resulting in the increasement of carrier mobility in the matrix [9]. Therefore, these two factors account for the decline of the DC resistivity with the increase of filler content.

It is worth mentioning that the extent of decline in Figure 4 varies with different liquid rubber, i.e., compared with the samples with HTPB and EHTPB, the introduction of CTBN and HTBN to epoxy resin has even smaller influences on the composite resistivity. This may be due to the different barriers and traps near the interfaces of two-phase structure in different composite materials investigated above. Those traps and barriers will impede the migration of carriers. However, this kind of obstructive effect is weak to change the decline trend of DC resistivity with the increase of filler content. By comparing the effects of four kinds of liquid rubber on DC resistance, we found that the stronger the polarity of liquid rubber, the smaller the decrease of matrix resistance. The barrier and trapping effect of the interface on carriers is closely related to the interfacial polarization, which will be studied in the following part, 5.2.2.

### 5.2. Analysis of Relative Permittivity and Dielectric Loss

#### 5.2.1. Relative Permittivity and Dielectric Loss at Different Rubber Contents

**Figure 5:**
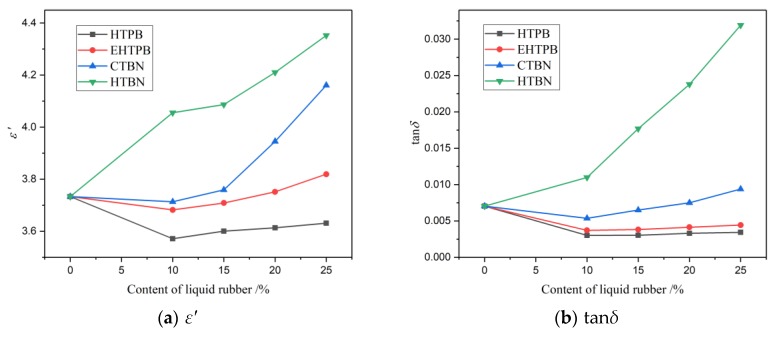
The *ε*′ and tan*δ* of epoxy resin matrix vary with the content of liquid rubber. (**a**) *ε*′; (**b**) tan*δ*. For the sample with HTBN, the *ε*′ and tan*δ* increase with the increase of filler content. For the sample with HTPB, EHTPB, and CTBN, the *ε*′ and tan*δ* decrease first and then increase with the increase of filler content.

In AC power equipment, the relative dielectric constant (*ε*′) and dielectric loss (tan*δ*) determine the potential distribution and loss heating in insulating materials [23]. The dielectric spectra of epoxy resins toughened by different liquid rubbers are measured at different temperatures. The *ε*′ and tan*δ* (50 Hz) of samples with different filler contents at room temperature (25 °C) are plotted in Figure 5.

According to Figure 5, *ε*′ and tan*δ* increase with the increase of molecular polarity of liquid rubber in general. For the samples with HTBN, the *ε*′ and tan*δ* increase more significantly with the filler content than the rest of the other samples. As a contrast, the *ε*′ and tan*δ* of samples with HTPB, EHTPB, and CTBN decrease initially with the increase of filler content and then start to increase when the filler content is reaching a certain value (around 10% to be exact). The lower values of *ε*′ and tan*δ* of insulating materials is more beneficial for their applications in the field of power equipment and electronic packaging.

The effect of liquid rubber with different polarity on dielectric loss and DC resistivity appears different due to both conductivity loss and polarization loss contributing to the dielectric loss. The conductivity loss is caused by the motion of carriers, while the polarization loss is the superposition of multiple relaxation processes. At room temperature, the polarization loss plays a dominant role in dielectric loss as the conductivity loss is way smaller, leading to the increasement of dielectric loss with liquid rubber polarity. The effect of rubber polarity on the polarization process will be discussed in detail below.

#### 5.2.2. Temperature Spectrum and Frequency Spectrum of Samples with Different Liquid Rubber

**Figure 6:**
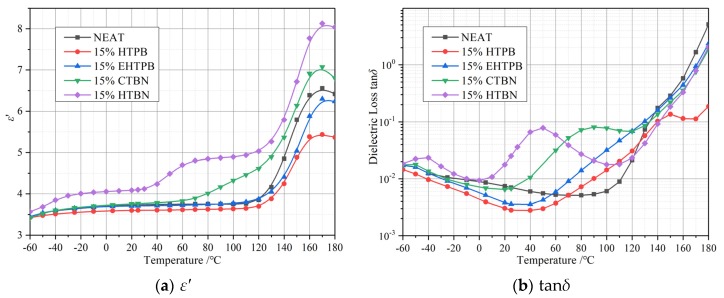
Temperature spectrum of epoxy resin toughened by four kinds of liquid rubber at 15% liquid rubber content (50 Hz). (**a**) *ε*′; (**b**) tan*δ*. *ε*′ increases with the increase of the polarity of liquid rubber. Relaxation peaks appear in the curves of tan*δ* due to interfacial polarization.

**Figure 7:**
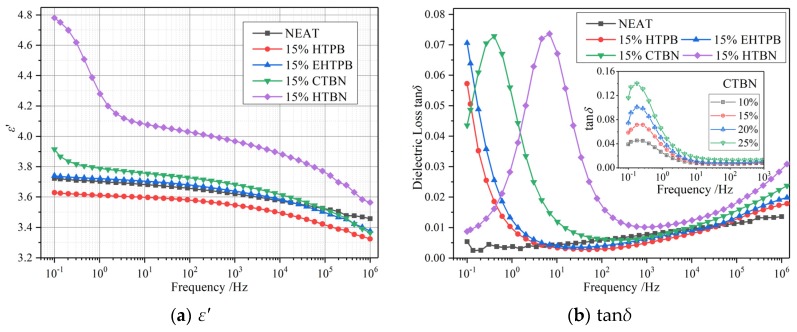
Frequency spectrum of epoxy resin toughened by four kinds of liquid rubber at 15% liquid rubber content (25 °C). (**a**) *ε*′; (**b**) tan*δ*. *ε*′ increases with the increase of the polarity of liquid rubber. Interfacial polarizations appear in the curves of tan*δ*.

In order to have a more thorough understanding of the influence of different liquid rubbers on the dielectric parameters of epoxy resin, the temperature spectrum and frequency spectrum are measured to explain the change of their relaxation behaviors. The temperature spectrum of 50 Hz is shown in Figure 6 and the frequency spectrum of 25 °C is shown in Figure 7.

In the temperature spectrum, the *ε*′ and tan*δ* of samples with HTPB and EHTPB fillers are lower than those of pure epoxy resin in a wide temperature range when the filler contents are smaller. For samples with CTBN fillers, *ε*′ and tan*δ* are smaller than those of pure epoxy resin before 30 °C. While for samples with HTBN fillers, ε′ and tanδ are higher than those of pure epoxy resin at all temperatures in our experiment.

At low temperatures, samples with HTBN have an obvious relaxation polarization process. Therefore, the *ε*′ and tan*δ* of samples with HTBN are larger than those of pure epoxy resin at all temperatures. However, no obvious polarization process is found for the other three kinds of samples in this low temperature range, thus no significant increasement of *ε*′ and tan*δ* could be noticed for these three kinds of samples below 30 °C and they are lower than those of pure epoxy resin.

*ε*′ of samples with HTBN and CTBN fillers starts to rise in the middle temperature range (20–100 °C approximately) and a peak value of tanδ could be found in their dielectric loss plot in this temperature range as well (50 °C for HTBN and 90 °C for CTBN, respectively). This indicates that a relaxation polarization is introduced by these two kinds of liquid rubbers. In addition, a relaxation polarization process could be found for all the samples when the temperature is higher and this is mainly caused by the α relaxation process of epoxy resin. Similar to samples with HTBN and CTBN, there should have been relaxation polarization processes for samples with HTPB and EHTPB fillers, as well in a medium temperature range. It is not recognizable in our plots due to the overlapping between relaxation polarization process and the α relaxation polarization process of epoxy resin in this temperature range.

According to the analysis above, epoxy resin toughened by HTPB and EHTPB can achieve lower dielectric constant and loss in a larger temperature range, which makes it easier to meet the strict *ε*′ and heating requirements. The relaxation process caused by CTBN and HTBN in the medium temperature range should be attributed to the interfacial polarization. This relaxation process leads to a maximum dielectric loss, resulting in an increasement of heat generation in this temperature range.

In frequency spectrum plots in Figure 7, a relaxation peak could be found on each dielectric loss curve after adding liquid rubber and move towards higher frequency with increase of the polarity of liquid rubbers. This relaxation peak indicates that the addition of liquid rubber introduces a new polarization process into the matrix, which is the same relaxation process as that occurs in the middle temperature range of temperature spectrum. Judging from the frequency value of the relaxation peak, this relaxation is caused by the interfacial polarization between rubber particles and epoxy matrix. It is worth mentioning that according to literature [10], the values of *ε*′ and tan*δ* lies for epoxy/liquid rubber composites in the corresponding temperature and frequency spectrum are smaller than those of pure epoxy resin, and no such relaxation peaks could be found based on our experimental results. This is mainly due to the selection of different types of liquid rubber and matrix, resulting in the different temperature and frequencies when this relaxation peak occurs. Additionally, the interfacial relaxation peaks have not been measured and investigated in their researches.

The interfacial polarizations of samples with CTBN and HTBN can be accomplished within 60 s, while that of HTPB and EHTPB takes longer. The charges gathered at the interface can hinder the movement of carriers, which leads to the different variation of the curves in Figure 4 of DC resistivity.

The relaxation times (*τ*) of interfacial polarizations are different because of the different *ε*′ and conductivity (*σ*) of rubber particles formed by different liquid rubber fillers. The relaxation times of the sample with HTBN and CTBN fillers with larger polarity are smaller, while that of HTPB and EHTPB with smaller polarity are longer. At the position where relaxation peak occurs, the loss tan*δ* of epoxy resin with filler is more than 10 times of that without filler. Dielectric loss is converted to heat energy. A sharp increase in loss will lead to a sharp increase in heating at the relevant frequency. Therefore, when choosing liquid rubbers as a filler for the composite insulating material, the value of frequency for this designed material in which the peak of dielectric loss lies should be carefully considered to be away from the range of the working frequency in order to reduce heat accumulation in service time for the safe operation of equipment.

### 5.3. Analysis of Relaxation Processes

#### 5.3.1. Interfacial Polarization

**Figure 8:**
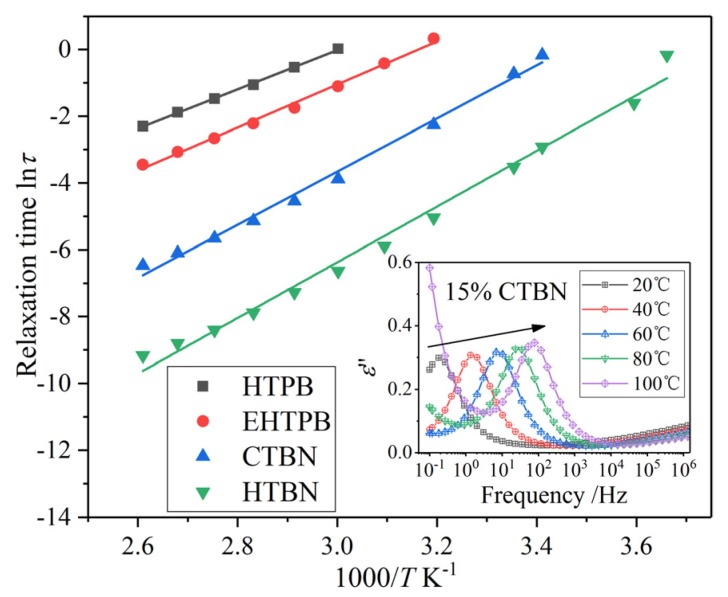
Relaxation time of interfacial polarization induced by different liquid rubber varies with temperature. The inset picture is the frequency spectrum of different temperature of sample with 15% CTBN, which shows the variation of the interfacial polarization.

The interfacial polarization will be introduced by adding different liquid rubbers to the epoxy matrix. The strength and relaxation time of interfacial polarization directly affect *ε*′ and tan*δ* of the composites. For epoxy resin toughened by a certain kind of liquid rubber, the strength of interfacial polarizations increases with the increase of filler content, while the relaxation time does not change much [16]. The variation of polarization relaxation peak introduced by CTBN filler at different contents is shown in the insert figure of Figure 7b. In order to analyze the interfacial polarization, the frequencies corresponding to interfacial polarization peaks at different temperatures are extracted and converted into relaxation time, and the variation of relaxation time with temperature is shown in Figure 8. The relaxation times increase with the temperature growth for all our investigated composite samples. The relaxation process of interfacial polarization generally conforms to the Arrhenius equation [16,24]. Thus, the Arrhenius equation is used to fit the data, and the results are shown in Table 1.

It can be seen from the determination coefficients that these interfacial polarization processes comply with this rule. Among the apparent activation energies (*E*_a_), samples with HTPB is the smallest while that of HTBN is the largest. The overall *E*_a_ increases with the increase of the polarity of liquid rubbers.

The *E*_a_ characterizes the difficulty of interfacial polarization. According to the fitting results of activation energy, the energy required for interfacial polarization of the samples with HTPB is the smallest. However, it can be seen from the curve of Figure 7 that the frequency of interfacial polarization corresponding to the sample with HTPB at the same temperature is lower than others. Therefore, the fitted activation energy is not consistent with the relaxation time distribution of interfacial polarization at 25 °C. This contradiction indicates that the apparent activation energy should have a deeper meaning.

The significance of *E*_a_ is further explored from the data in Figure 8. The slopes of the four lines in Figure 8 are different, which will lead to the intersection of lines in the low temperature region. The relaxation time of interfacial polarization varies with temperature mainly due to the temperature dependence of material parameters on both sides of the interface [25]. The main factors affecting the relaxation time are the *σ* and *ε*′ of the materials on both sides of the interface. The increase of temperature will lead to the increase of the *σ* and *ε*′. Since the variation of *ε*′ with temperature is much smaller than that of *σ*, the relaxation time of interfacial polarization is mainly affected by the change of *σ* with temperature. For the four kinds of composites toughened by liquid rubber fillers, the conductivities of epoxy matrix are approximately the same. Therefore, the relaxation time of interfacial polarization is mainly affected by the temperature dependence of conductivity of the rubber phase. The electric conduction process of polymer itself conforms to the Arrhenius equation [26]. Therefore, the calculated apparent activation energy should mainly reflect conductivity variation of the rubber phase with temperature. The possible intersection in the low temperature range in Figure 8 is attributed to the similar value of the parameters of rubber phase in this temperature range. With the increase of temperature, the growth rate of conductivity is different, among which that of samples with HTBN is the fastest, while that of HTPB is the slowest. That is to say, for the rubber phase formed by the four kinds of liquid rubbers, the greater the polarity of the molecule is, the faster the conductivity increases with temperature. This is because the directional motion of dipole moments in polar molecules grows stronger with the increase of temperature, which is more conducive to carrier migration.

The appearance of interfacial polarization increases the *ε*′ and finally leads to a maximum of dielectric loss. Through the analysis above, it is found that the temperature and frequency ranged for the occurrence of interfacial polarization can be controlled by choosing the liquid rubber with reasonable polarity and the variation rate of interfacial polarization with temperature can also be manipulated likewise. Finally, the *ε*′ and tan*δ* of epoxy/liquid rubber composites can be adjusted to the required value to some extent by choosing the right liquid rubber filler with a certain polarity.

#### 5.3.2. Relaxation Processes in the Low Temperature Range

**Figure 9:**
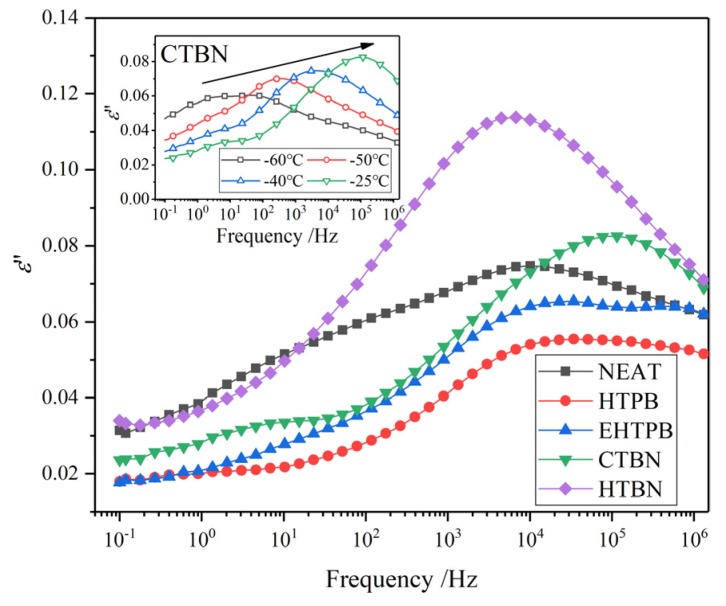
Spectrum curves of epoxy resins toughened by a different rubber at −25 °C. The inset picture is the frequency spectrum of low temperatures of sample with 15% CTBN, which shows the variety of the interfacial polarization. The relaxation process on pure epoxy resin curve is the secondary transition relaxation process of epoxy resin. The relaxation process on the remaining curves is mainly caused by the *α* transition of rubber molecules.

The relaxation processes in the low temperature range determine the *ε*′ and tan*δ* of materials at room temperature. The dielectric spectrum of samples with four kinds of liquid rubbers at low temperature are extracted and the corresponding dielectric loss factor (*ε*″) curves are plotted in Figure 9.

The curves of *ε*″ at the temperature of −25 °C show that an obvious relaxation process appears before and after adding liquid rubber. Previous studies have found that the addition of liquid rubber leads to a relaxation polarization process in low temperature range, which is caused by the α transition of rubber molecules [16]. The strength of α transition of rubber molecules and secondary transition of epoxy resin directly affect the *ε*′ and tan*δ* of composites at the temperature above.

The addition of liquid rubber affects the secondary transition strength of epoxy resin through two ways. On the one hand, the presence of rubber particles will increase the number of branched and side chains of cross-linked epoxy resin. On the other hand, the addition of liquid rubber reduces the number of epoxy resin molecules in the unit volume. Therefore, the secondary transition strength of epoxy resin decreases due to the combination of these two effects. This explanation can be confirmed by Figure 9. It shows that the values of the *ε*″ at low frequency are smaller than those of pure epoxy resin and increase with the increase of the polarity of rubber molecules. In the high frequency range, although the relaxation peaks caused by the α relaxation process of rubber molecules overlap with the secondary transition peaks of epoxy resin, it can be seen that the strength of relaxation peaks increases with the increase of the polarity of rubber molecules.

The relaxation peak strength of samples with HTPB and EHTPB in low temperature range is smaller than that of pure epoxy resin, resulting in the lower *ε*′ and tan*δ* in the temperature spectrum curve of Figure 6. The relaxation peak strength of samples with CTBN and HTBN is also consistent with the results of temperature spectrum. Therefore, in order to obtain smaller *ε*′ and tan*δ*, for composites material, the liquid rubber with smaller polarity should be chosen.

## 6. Conclusions

Four kinds of liquid rubber with different polarity have been used to toughen epoxy resin. The effects of liquid rubbers with different polarity on the dielectric properties including DC resistivity, relative permittivity and dielectric loss, along with the relaxation characteristics of epoxy resin are measured and analyzed. We found that the addition of liquid rubber mainly affects the relaxation characteristics in the low temperature range, as well as the interfacial polarization between the liquid rubber and epoxy resin. Lower dielectric loss could be obtained for the epoxy composites when liquid rubber with lower polarity was added to the composite material. By increasing of the polarity of liquid rubbers, the relative dielectric constant and dielectric loss of the corresponding composites increases accordingly.

The α relaxation of rubber molecule is introduced after the addition of liquid rubber to epoxy resin when the temperature is relatively lower and the strength of relaxation increases with the increase of the polarity of rubber molecules. For the interfacial polarization between rubber phase and epoxy matrix, the apparent activation energy fitted by the Arrhenius plot increases with the increase of rubber polarity. Through further analysis, we found that the apparent activation energy is the reflection and indicator of how difficult the conduction process of rubber phase is.

## Figures and Tables

**Table 1:** Apparent activation energy of the interfacial polarization.

Types of Liquid Rubber (15%)	Apparent Activation Energy *E*_a_/kJ·mol^−1^	Determination Coefficient
HTPB	48.8	0.999
EHTPB	53.6	0.994
CTBN	66.1	0.992
HTBN	69.3	0.988
